# Folic acid delays age-related cognitive decline in senescence-accelerated mouse prone 8: alleviating telomere attrition as a potential mechanism

**DOI:** 10.18632/aging.102461

**Published:** 2019-11-22

**Authors:** Xin Lv, Xinyan Wang, Yalan Wang, Dezheng Zhou, Wen Li, John X. Wilson, Hong Chang, Guowei Huang

**Affiliations:** 1 Department of Nutrition and Food Science, School of Public Health, Tianjin Medical University, Tianjin 300070, China; 2 Tianjin Key Laboratory of Environment, Nutrition and Public Health, Center for International Collaborative Research on Environment, Nutrition and Public Health, Tianjin 300070, China; 3 Department of Exercise and Nutrition Sciences, School of Public Health and Health Professions, University at Buffalo, Buffalo, NY 14214, USA; 4 Department of Rehabilitation Medicine, Tianjin Medical University, Tianjin 300070, China

**Keywords:** folic acid, cognitive decline, neurodegeneration, telomere attrition, mitochondrial dysfunction

## Abstract

The occurrence of telomere attrition in brain may cause senescence and death of neurons, leading to cognitive decline. Folic acid (FA) has been reported to improve cognitive performance in mild cognitive impairment; however, its association with telomere remains unclear. The study aimed to investigate if alleviation of telomere attrition by FA supplementation could act as a potential mechanism to delay age-related cognitive decline in senescence-accelerated mouse prone 8 (SAMP8). Aged SAMP8 mice were assigned to four treatment groups: FAdeficient diet (FA-D) group, FA-normal diet (FA-N) group, low FA-supplemented diet (FA-L) group and high FAsupplemented diet (FA-H) group. There was also an age-matched senescence-accelerated mouse resistant 1 (SAMR1) control group (Con-R), and a young SAMP8 control group (Con-Y). The results demonstrated that FA supplementation delayed age-related cognitive decline and neurodegeneration in SAMP8 mice. Importantly, this effect could be attributed to the alleviated telomere attrition, which might be interpreted by the decreased levels of reactive oxygen species. Additionally, improved telomere integrity stimulated mitochondrial function via telomere-p53-mithondria pathway, consequently delayed neuronal degeneration. In conclusion, we demonstrate that FA supplementation delays age-related neurodegeneration and cognitive decline in SAMP8 mice, in which alleviated telomere attrition could serve as one influential factor in the process.

## INTRODUCTION

The incidence of neurodegenerative disorders including Alzheimer’s disease (AD) is increasing rapidly with the extension of average life expectancy [1]. It has been suggested that brain senescence may contribute to AD pathogenesis, and represent a link between aging process and disease development [2]. Brain aging is characterized by cognitive decline [3] with neuronal dysfunction that manifests as lost in connection [4] and altered signaling pathways and neurotransmitter systems [5].

Folate is important for the functioning of the nervous system at all ages [6]. It provides nucleotide precursors and maintains homocysteine (Hcy) at non-toxic levels through one-carbon metabolism [7]. Folate deficiency causes hyperhomocysteinemia, excessive levels of reactive oxygen species (ROS), and DNA damage [8–10]. Brain neurons become susceptible to ROS-induced DNA damage if antioxidants are depleted [11]. Perhaps through this mechanism, transgenic mouse models of AD exhibited increased cellular DNA damage and hippocampal neurodegeneration when maintained on a folic acid (FA)-deficient diet [12]. In the general population, poor cognitive performance is associated with low serum folate concentration [13], whereas FA supplementation appears to improve cognitive function especially in people with mild cognitive impairment [14, 15].

Telomere, which consists of a tract of tandemly repeated short DNA sequence (TTAGGG) bound by shelterin (a protective protein complex), is located at chromosome ends and acts to maintain chromosomal integrity and stability [16]. Telomere shortening occurs with cell division and degrading activities; however, it can be counteracted by reverse transcriptase telomerase through synthesizing and adding the new telomeric DNA to chromosome ends [17, 18]. Late generation telomerase-deficient mice showed neuronal loss in the hippocampal region and frontal cortex associated with short-term memory deficits [19], impaired olfaction [20], and anxiety-like behaviors [21], while telomerase reactivation in adult mice was able to delay and reverse age-related decline in cognitive performance [20], indicating that telomere stability is required for normal brain function. Telomeric DNA is especially susceptible to ROS damage owing to its 5'-GGG-3' repeat sequence, and its single stranded 3' overhang makes repair less efficient than coding regions and may result in telomere shortening [22–24]. Eventually telomeres shorten so much that they do not protect chromosomes from degradation, leading to cell senescence and death [25]. Some epidemiological studies have revealed that peripheral blood leukocyte telomere length may correlate with serum folate concentration [13–15].

We hypothesis that alleviation of telomere attrition by FA supplementation could act as a potential mechanism to delay age-related neurodegeneration and cognitive decline in senescence-accelerated mouse prone 8 (SAMP8). Cognitive decline was evaluated by Morris water maze (MWM) and open field (OF) tests, whereas neurodegeneration was assessed by hematoxylin and eosin (HE), Nissl, and Fluoro-Jade B (FJB) staining. Additionally, to investigate FA’s mechanism of action, H_2_O_2_ concentration, hydroxyl free radical (HO^**·**^) suppression ability and superoxide anion free radical (O_2_^**· –**^) suppression ability were measured to assess ROS levels, telomeric DNA oxidation and telomere length were measured to assess telomere attrition, and mitochondrial DNA (mtDNA) copy number, mtDNA deletions and ATP level were measured to assess mitochondrial biogenesis and function.

## RESULTS

### Dietary FA supplementation raised folate concentrations and decreased age-related ROS levels in SAMP8 mice

Aged SAMP8 mice were assigned to four treatment groups: FA-deficient diet (FA-D) group, FA-normal diet (FA-N) group, low FA-supplemented diet (FA-L) group, and high FA-supplemented diet (FA-H) group. There were also an age-matched senescence-accelerated mouse resistant 1 (SAMR1) control group (Con-R) and a young SAMP8 control group (Con-Y), both of which were fed with FA-normal diet.

**Figure 1:**
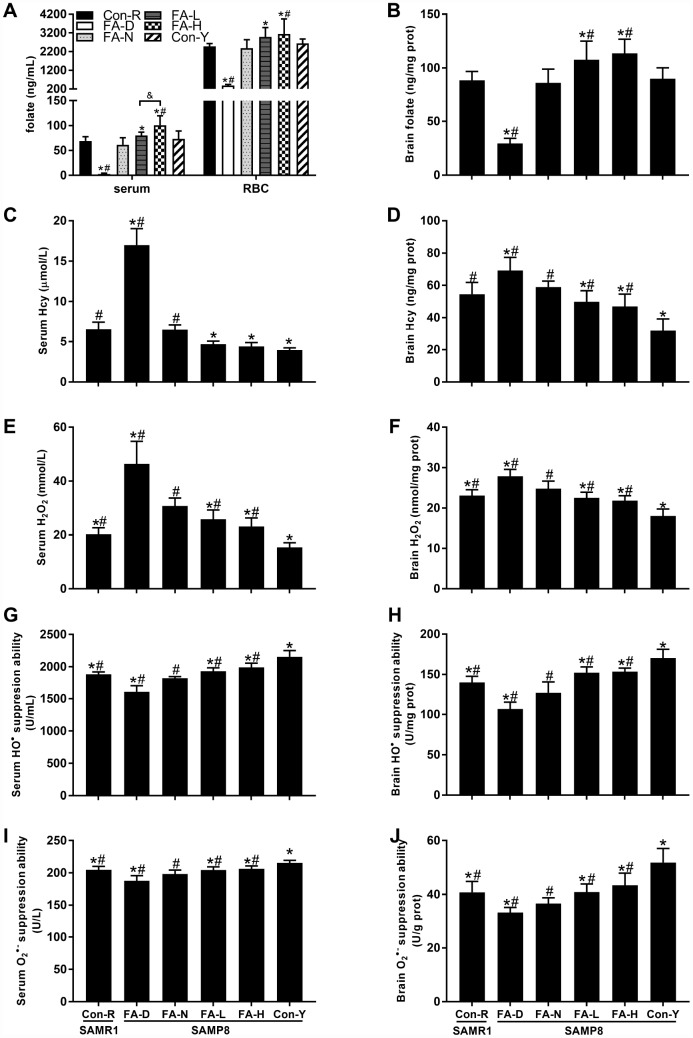
**Dietary folic acid (FA) supplementation raised folate concentrations and decreased reactive oxygen species (ROS) levels in Senescence Accelerated Mouse Prone 8 (SAMP8) mice.** Aged SAMP8 mice were assigned to four treatment groups: FA-deficient diet (FA-D) group, FA-normal diet (FA-N) group, low FA-supplemented diet (FA-L) group, and high FA-supplemented diet (FA-H) group. There were also an age-matched senescence-accelerated mouse resistant 1 (SAMR1) control group (Con-R), and a young SAMP8 control group (Con-Y), both of which were fed with FA-normal diet. Folate concentrations were detected in serum [*F*_(5,54)_ = 65.507, *P*<0.001], red blood cell (RBC) [*F*_(5,54)_ = 39.041, *P*<0.001] (**A**) and brain [*F*_(5,54)_ = 64.878, *P*<0.001] (**B**). Hcy concentrations were detected in serum [*F*_(5,54)_ = 250.012, *P*<0.001] (**C**) and brain [*F*_(5,54)_ = 29.529, *P*<0.001] (**D**). ROS levels were indicated by H_2_O_2_ levels in serum [*F*_(5,54)_ = 64.322, *P*<0.001] (**E**) and brain [*F*_(5,54)_ = 44.100, *P*<0.001] (**F**), HO^•^ suppression abilities in serum [*F*_(5,54)_ = 71.992, *P*<0.001] (**G**) and brain [*F*_(5,54)_ = 65.702, *P*<0.001] (**H**), and O_2_^• –^ suppression abilities in serum [*F*_(5,54)_ = 26.796, *P*<0.001] (**I**) and brain [*F*_(5,54)_ = 30.031, *P*<0.001] (**J**). Data are expressed as mean ± standard deviation (SD) (n= 10 mice/group). *P<0.05 compared with FA-N group. ^#^*P*<0.05 compared with Con-Y group. ^&^*P*<0.05 compared between FA-L and FA-H groups.

FA-N, Con-R and Con-Y groups were all fed with FA-normal diet, and there is no significant difference in folate (Figure 1A and 1B) or Hcy (Figure 1C and 1D) concentrations among them. Among the 4 aged SAMP8 treatment groups, folate concentrations in serum, red blood cells (RBC) and brain were increased by dietary FA supplementation and decreased by dietary FA deficiency (*P*<0.05, Figure 1A and 1B). Comparison of the two FA supplementation groups showed the FA-H group had a higher folate concentration in serum (*P*<0.05, Figure 1A), but no significant difference between those groups were found for the folate concentrations in RBC and brain (Figure 1A and 1B).

The FA deficient diet increased Hcy concentrations in serum and brain, whereas FA supplementation decreased Hcy concentration in brain of aged SAMP8 mice (*P*<0.05, Figure 1C and 1D). Hcy concentrations did not differ significantly between the FA-L and FA-H groups (Figure 1C and 1D).

An effect of age on increasing ROS levels was evident in the data showing that, in serum and brain, all 4 of the aged SAMP8 groups had higher H_2_O_2_ concentration, and lower HO^**•**^ suppression ability and O_2_^**• –**^ suppression ability than the Con-Y group did (*P*<0.05, Figure 1E–1J). Among the 4 aged SAMP8 treatment groups, FA deficiency increased H_2_O_2_ level and decreased HO^**•**^ and O_2_^**• –**^ suppression abilities, whereas FA supplementation decreased H_2_O_2_ level and increased HO^**•**^ and O_2_^**• –**^ suppression abilities (*P*<0.05, Figure 1E–1J). There were no significant differences between the FA-L and FA-H groups (Figure 1E–1J).

Taken together, folate concentrations in serum, RBC and brain were raised in response to dietary FA supplementation and decreased by FA deficiency. Ten-month-old SAMP8 mice exhibited age-related elevation of ROS levels in serum and brain that was increased by FA deficiency but decreased by FA supplementation.

### FA supplementation delayed age-related cognitive decline in SAMP8 mice

**Figure 2:**
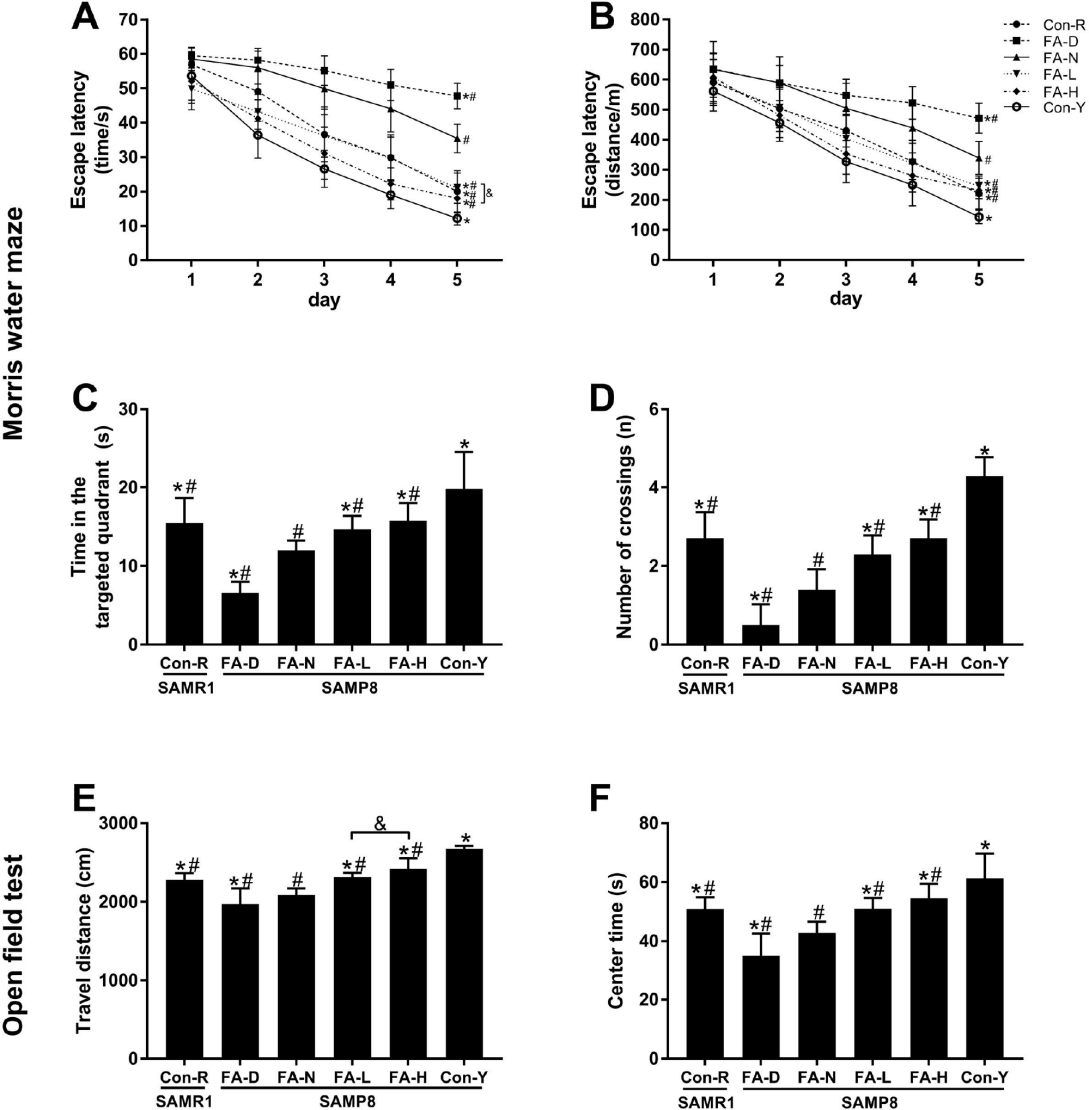
**FA supplementation improved cognitive performances in SAMP8 mice.** Mice were assigned to treatment groups as described in Figure 1. Cognitive performances were evaluated by the Morris water maze (MWM) (**A**–**D**) and open field (OF) (**E**–**F**) tests. Escape latency indicated in time [*F*_time_ = 383.018, *P*<0.001; *F*_treatment_ = 56.650, *P*<0.001] (**A**) and distance [*F*_time_ = 363.834, *P*<0.001; *F*_treatment_ = 27.208, *P*<0.001] (**B**) during the spatial acquisition phase of the MWM test. Time in the targeted quadrant [*F*_(5,54)_ = 26.253, *P*<0.001] (**C**) and number of crossings [*F*_(5,54)_ = 59.071, *P*<0.001] (**D**) during the spatial probe phase of the MWM test. (**E**) Total travel distance that the mouse moved in the OF test [*F*_(5,54)_ = 48.038, *P*<0.001]. (**F**) Time spent in the center of the OF [*F*_(5,54)_ = 25.850, *P*<0.001]. Data are expressed as mean ± SD (n= 10 mice/group). *P<0.05 compared with FA-N group. ^#^*P*<0.05 compared with Con-Y group. ^&^*P*<0.05 compared between FA-L and FA-H groups.

The MWM test was performed to evaluate spatial learning and memory ability. Escape latency decreased (i.e. learning ability increased) as the days of trial increased in all 6 groups of mice. Repeated-measures ANOVA showed that the FA-N group required longer time and distance to find the platform than the Con-R group did (*P*<0.05, Figure 2A and 2B), indicating that SAMP8 mice fed a FA-normal diet exhibited accelerated learning decline at 10 months old. All 4 of the aged SAMP8 groups took longer time and distance to find the platform than did the Con-Y group (*P*<0.05, Figure 2A and 2B), indicating that aging caused learning ability to decline. Among the 4 aged SAMP8 treatment groups, FA deficiency increased escape latency and FA supplementation decreased escape latency, as compared to the FA-N group (*P*<0.05, Figure 2A and 2B), which provided further evidence that FA supplementation delayed age-related learning decline in SAMP8 mice.

The MWM test’s results also showed that FA-N group spent shorter time in the targeted quadrant and had few number of crossings as compared to Con-R group, and the same tendency were found in all 4 of the aged SAMP8 groups as compared to Con-Y group, indicating an age-related memory decline in 10-month-old SAMP8 mice (*P*<0.05, Figure 2C and 2D). Among the 4 aged SAMP8 treatment groups, FA deficiency decreased time in the targeted quadrant and number of crossings, and FA supplementation increased time in the targeted quadrant and number of crossings, as compared to the FA-N group (*P*<0.05, Figure 2C and 2D). Comparison of those two FA supplementation groups showed FA-H group had decreased escape latency in time (*P*<0.05, Figure 2A), but no significant difference between the groups was found for time in the targeted quadrant or number of crossings (Figure 2C and 2D).

OF test was performed to measure the locomotor activity and exploratory behavior in a novel environment. The results of the OF test showed that the FA-N group had shorter travel distance and spent less time in the center than the Con-R group did, and the same tendency were found in all 4 of the aged SAMP8 groups compared to the Con-Y group, indicating that age-related locomotor activity declined in 10-month-old SAMP8 mice (*P*<0.05, Figure 2E and 2F). Among the 4 aged SAMP8 treatment groups, FA deficiency decreased travel distance and center time, and FA supplementation increased travel distance and center time, compared to the FA-N group (*P*<0.05, Figure 2E and 2F). Comparison of those two FA supplementation groups showed the FA-H group had farther travel distance (*P*<0.05, Figure 2E), but no significant difference between the groups was found for center time (Figure 2F).

Additionally, the mice were weighted every week during the experiment, and there is no significant difference in changes of body weight among the groups (*P*>0.05), thereby excluded the confounding effects of weight loss on cognitive function.

Taken together, the results showed that age-related cognitive decline appeared in 10-month-old SAMP8 mice, and that impairment was accelerated by FA deficiency. FA supplementation, especially at the high level, delayed age-related cognitive decline.

### FA supplementation delayed age-related neurodegeneration in cerebral cortex and hippocampal CA1 region of SAMP8 mice

**Figure 3:**
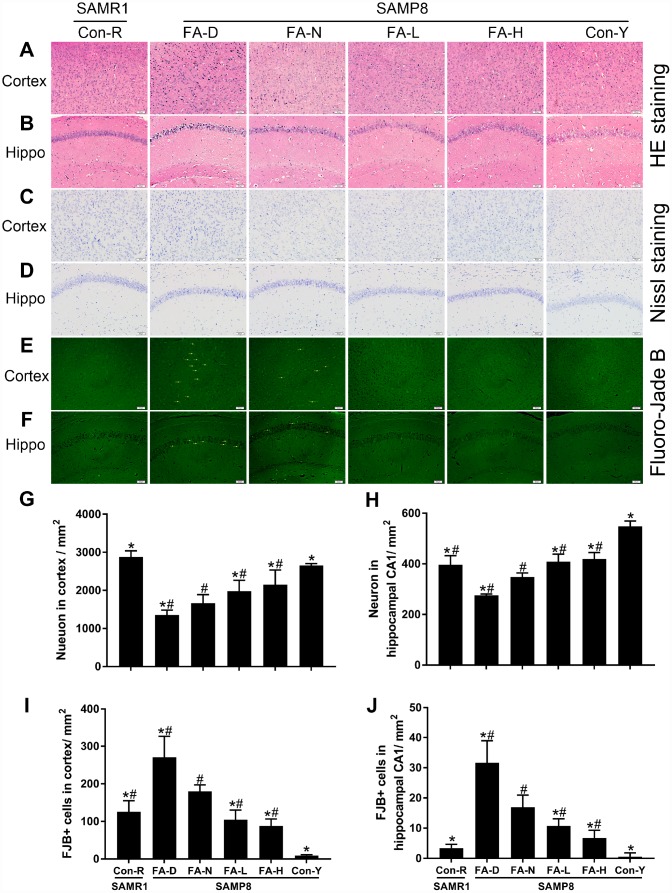
**FA supplementation delayed neurodegeneration and stimulated neuronal survival in cerebral cortex and hippocampal CA1 region in SAMP8 mice.** Mice were assigned to treatment groups as described in Figure 1. Representative micrographs of HE staining in cerebral cortex (**A**) and hippocampal CA1 region (**B**), Nissl staining in cerebral cortex (**C**) and hippocampal CA1 region (**D**), Fluoro-Jade B (FJB) staining in cerebral cortex (**E**) and hippocampal CA1 region (**F**). Quantitative analysis of surviving neurons indicated by Nissl staining in cerebral cortex [*F*_(5,24)_ = 30.882, *P*<0.001] (**G**) and hippocampal CA1 region [*F*_(5,24)_ = 68.256, *P*<0.001] (**H**), and degenerated neurons indicated by FJB-positive cells in cerebral cortex [*F*_(5,24)_ = 44.797, *P*<0.001] (**I**) and hippocampal CA1 region [*F*_(5,24)_ = 45.630, *P*<0.001] (**J**). Yellow arrow indicates FJB positive cell. Scale bar = 50 μm. Data are expressed as mean ± SD (n= 5 mice/group). *P<0.05 compared with FA-N group. ^#^*P*<0.05 compared with Con-Y group.

HE staining was performed to observe neuronal morphology in the cerebral cortex and hippocampal CA1 region. In the Con-R and Con-Y groups, the majority of the neurons contained abundant cytoplasm with well-defined nuclei. In the aged SAMP8 groups, more of the neurons had condensed nuclei chromatin. The senescent changes were most severe in the FA-D group and were partly prevented by FA supplementation (Figure 3A and 3B).

Nissl-staining and FJB-staining were performed to quantify neuronal survival and degeneration, respectively. The FA-N group had fewer surviving neurons and more degenerated neurons in the cerebral cortex and hippocampal CA1 region than the Con-R group did. The same changes were found in all 4 of the aged SAMP8 groups compared to the Con-Y group (*P*<0.05, Figure 3C–3J), indicating age-related neurodegeneration in 10-month-old SAMP8 mice. Among the 4 aged SAMP8 treatment groups, FA-D group had the fewest surviving neurons and the most degenerated neurons, and FA supplementation increased neuronal survival and decreased neuronal degeneration (*P*<0.05, Figure 3C–3J). There were no significant differences between the FA-L and FA-H groups (Figure 3C–3J).

Taken together, the results showed that 10-month-old SAMP8 mice exhibited age-related neurodegeneration in cerebral cortex and hippocampal CA1 region, and that neurodegeneration was worsened by FA deficiency and partly prevented by FA supplementation.

### FA supplementation alleviated age-related telomere attrition in brain of SAMP8 mice

**Figure 4:**
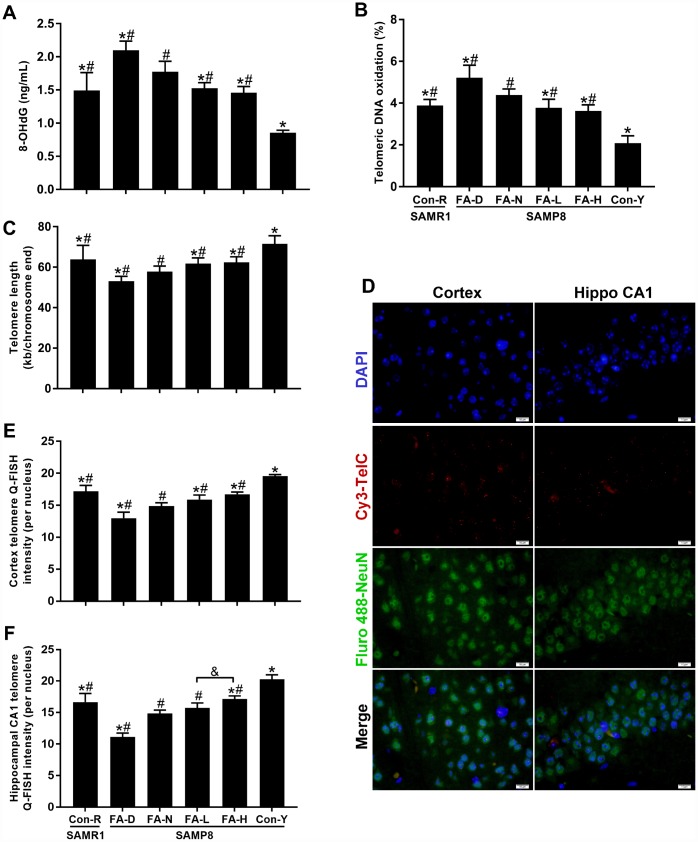
**FA supplementation protected against telomeric DNA oxidation and telomere length shortening in brain of SAMP8 mice.** Mice were assigned to treatment groups as described in Figure 1. (**A**) Level of 8-hydroxy-2'-deoxyguanosine (8-OHdG) in genomic DNA quantified by ELISA [*F*_(5,54)_ = 75.370, *P*<0.001]. (**B**) Level of telomeric DNA oxidation quantified by qPCR [*F*_(5,54)_ = 70.139, *P*<0.001]. (**C**) Telomere length quantified by qPCR [*F*_(5,54)_ = 24.184, *P*<0.001]. (**D**) Representative telomere Q-FISH images in neurons (blue DAPI-stained nuclei; red telomeric probe; green NeuN-stained neuron). Neuronal telomere length quantified by Q-FISH in cerebral cortex [*F*_(5,24)_ = 54.582, *P*<0.001] (**E**) and hippocampal CA1 region [*F*_(5,24)_ = 68.765, *P*<0.001] (**F**). Scale bar = 10 μm. Data are expressed as mean ± SD (n=10 mice/group for ELISA and qPCR assays, n=5 mice/group for Q-FISH assay). *P<0.05 compared with FA-N group. ^#^*P*<0.05 compared with Con-Y group. ^&^*P*<0.05 compared between FA-L and FA-H groups.

Telomeric DNA oxidation and telomere length were measured to determine if FA affected age-related telomere attrition. FA-N group had more 8-OHdG in genomic and telomeric DNA than the Con-R group did. The same changes were found in all 4 of the aged SAMP8 groups compared to the Con-Y group, which evident a marked effect of age (*P*<0.05, Figure 4A and 4B). Among the 4 aged SAMP8 treatment groups, the levels of 8-OHdG in genomic and telomeric DNA were increased by FA deficiency and decreased by FA supplementation, compared to the FA-N group (*P*<0.05, Figure 4A and 4B). There was no significant difference between the FA-L and FA-H groups (Figure 4A and 4B).

The results of qPCR showed that the FA-N group had shorter telomere length than the Con-R group did, and the same change was found in all 4 of the aged SAMP8 groups compared to Con-Y group (*P*<0.05, Figure 4C), indicating that age-related telomere shortening occurred in 10-month-old SAMP8 mice. Among the 4 aged SAMP8 treatment groups, FA deficiency accelerated telomere shortening, and FA supplementation delayed telomere shortening, compared to the FA-N group (*P*<0.05, Figure 4C). The Q-FISH assay performed on neurons (NeuN-positive cells) in the cerebral cortex and hippocampal CA1 region further confirmed the effects of FA intake on telomere shortening (*P*<0.05, Figure 4D–4F). Moreover, comparison of the Q-FISH assay results for the two FA supplementation groups showed that telomere shortening in neurons of the hippocampal CA1 region was alleviated more in the FA-H group (Figure 4E and 4F).

Taken together, the results showed that the brains of 10-month-old SAMP8 mice exhibited telomeric DNA oxidation and telomere length shortening, indicating age-related telomere attrition. Telomere attrition was increased by FA deficiency and alleviated by FA supplementation. Therefore, FA supplementation may prevent neurodegeneration in aged SAMP8 mice by alleviating telomere attrition.

### FA supplementation delayed age-related mitochondrial dysfunction through a telomere–p53–mitochondria pathway in brain of SAMP8 mice

**Figure 5:**
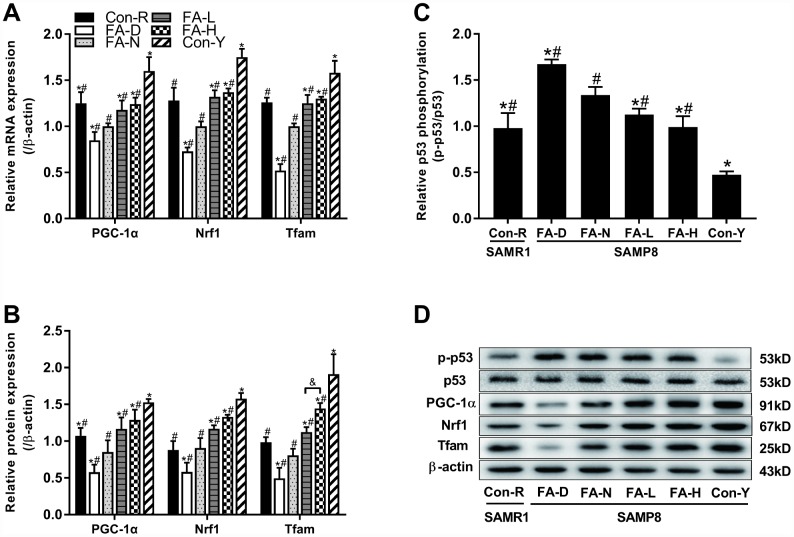
**FA supplementation regulated telomere–p53–mitochondria pathway in brain of SAMP8 mice.** Mice were assigned to treatment groups as described in Figure 1. (**A**) The relative mRNA levels of PGC-1α, Nrf1 and Tfam quantified by qPCR were normalized to β-actin and expressed as fold changes relative to the FA-N group [*F*_(5,54)_ = 30.274, *P*<0.001; *F*_(5,54)_ = 36.428, *P*<0.001; *F*_(5,54)_ = 34.239, *P*<0.001]. (**B**) The protein expressions of PGC-1α, Nrf1 and Tfam quantified by western blot were normalized to β-actin [*F*_(5,12)_ = 21.689, *P*<0.001; *F*_(5,12)_ = 39.452, *P*<0.001; *F*_(5,12)_ = 38.230, *P*<0.001]. (**C**) The level of phospho-p53 quantified by western blot was normalized to p53 [*F*_(5,12)_ = 51.308, *P*<0.001]. (**D**) Representative western blot of phospho-p53, p53, PGC-1α, Nrf1, Tfam and β-actin. Data are expressed as mean ± SD (n= 10 mice/group for qPCR, and n= 3 mice/group for western blot). *P<0.05 compared with FA-N group. ^#^*P*<0.05 compared with Con-Y group. ^&^*P*<0.05 compared between FA-L and FA-H groups.

We investigated whether telomere attrition could act through a telomere–p53–mitochondria pathway to trigger mitochondrial dysfunction and if the pathway was sensitive to FA. mRNA and protein expressions of three mitochondrial biogenesis-related genes, namely, peroxisome proliferator-activated receptor γ coactivator 1α (PGC-1α), nuclear respiratory factor 1 (Nrf1) and mitochondrial transcription factor A (Tfam) were quantified by qPCR and western blot assays. Additionally, phosphorylation level at Ser15 of p53 protein was also quantified by western blot. Results showed that all 4 of the aged SAMP8 groups had increased phospho-p53 levels, and decreased PGC-1α, Nrf1 and Tfam levels, compared to the Con-Y group (*P*<0.05, Figure 5A–5D), indicating that a telomere–p53–mitochondria pathway is altered by age in SAMP8 mice. Among the 4 aged SAMP8 treatment groups, the FA-D group had the highest phospho-p53 level and the lowest PGC-1α, Nrf1, Tfam levels (*P*<0.05, Figure 5A–5D). FA supplementation decreased phospho-p53 and increased PGC-1α, Nrf1, Tfam expression (*P*<0.05, Figure 5A–5D). Comparison of the two FA supplementation groups showed that Tfam protein expression was increased in the FA-H group compared to the FA-L group (Figure 5A–5D).

**Figure 6:**
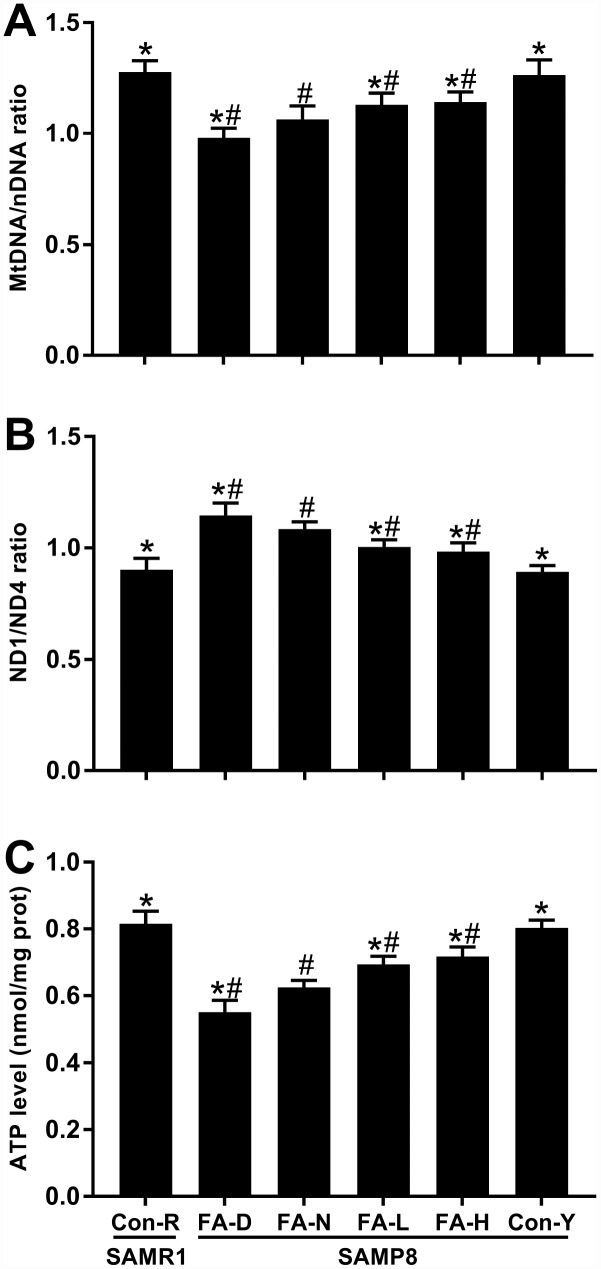
**FA supplementation improved mitochondrial biogenesis and function in brain of SAMP8 mice.** Mice were assigned to treatment groups as described in Figure 1. (**A**) Relative mitochondrial DNA copy number indicated by mtDNA/nDNA ratio [*F*_(5,54)_ = 43.946, *P*<0.001]. (**B**) Mitochondrial DNA deletions indicated by ND1/ND4 ratio [*F*_(5,54)_ = 59.644, *P*<0.001]. (**C**) Mitochondrial function indicated by ATP level [*F*_(5,54)_ = 123.771, *P*<0.001]. Data are expressed as mean ± SD (n= 10 mice/group). *P<0.05 compared with FA-N group. ^#^*P*<0.05 compared with Con-Y group.

The mitochondrial DNA/nuclear DNA (mtDNA/nDNA) ratio, ND1/ND4 ratio and ATP content were measured, respectively, to determine the relative mtDNA copy number, mtDNA deletions and mitochondrial function. The FA-N group had lower mtDNA/nDNA ratio, higher ND1/ND4 ratio and lower ATP level than did the Con-R group (*P*<0.05, Figure 6A–6C). All 4 of the aged SAMP8 groups exhibited the same changes in mitochondria compared to the Con-Y group, indicating impaired mitochondrial biogenesis and function are associated with age (*P*<0.05, Figure 6A–6C). FA supplementation partly prevented those impairments, whereas the FA-D group of aged SAMP8 mice had the lowest mtDNA/nDNA ratio, highest ND1/ND4 ratio and lowest ATP level (*P*<0.05, Figure 6A–6C). There were no significant differences between the FA-L and FA-H groups (Figure 6A–6C).

Taken together, the results showed that in SAMP8 mice there occurred age-related alterations in a telomere–p53–mitochondria pathway and mitochondrial function in brain, which were worsened by FA deficiency and improved by FA supplementation.

## DISCUSSION

The present study provides novel evidence that alleviation of telomere attrition by FA supplementation could act as a potential mechanism to delay age-related cognitive decline in SAMP8 mice. This evidence was obtained from experiments with the SAMP8 mouse, which is a model of age-related cognitive decline that exhibits early-stage neurodegeneration with impaired learning and memory abilities [26]. SAMP8 mice start aging rapidly at 4 months of age, although at younger ages they are identical to SAMR1 mice [27]. Therefore, we studied aged SAMP8 mice at 10 months old that had begun FA treatments at 4 months old. The FA treatments were FA-deficient (FA-D) diet, FA-normal (FA-N) diet, low FA-supplemented (FA-L) diet, and high FA-supplemented (FA-H) diet. Age-matched SAMR1 mice (Con-R) fed with FA-N diet formed a control group that experienced normal aging. Comparisons also were made with a group of younger SAMP8 mice fed with FA-N diet (Con-Y).

Comparison between the FA-N, Con-R and Con-Y groups confirmed that aged (10-month-old) SAMP8 mice had similar folate levels but worse cognitive performance, more deteriorative neurodegeneration, telomere attrition and mitochondrial dysfunction than did either age-matched SAMR1 mice or young (4-month-old) SAMP8 mice. However, the FA-L and FA-H groups showed improvements in learning/memory ability and locomotor activity, had few degenerated neurons and more surviving neurons in cerebral cortex and hippocampal CA1 region, as compared to the FA-N group, indicating that FA supplementation delayed age-related cognitive decline and neurodegeneration in SAMP8 mice. This protective effect of FA could be attributed to the alleviated telomere attrition that manifested as reduced telomeric DNA oxidation and telomere length shortening, and that alleviation might be interpreted by the decreased levels of ROS. Additionally, improved telomere integrity stimulated mitochondrial biogenesis and function via a telomere-p53-mithondria pathway.

Folate concentrations in serum, RBC and brain of aged SAMP8 mice rose in response to dietary FA supplementation. The FA-N diet (AIN-93M) contained 2.0 mg FA/kg**•**diet that was considered to meet the general nutritional requirements of mice [28]. The supplemented diets contained 2.5 and 3.0 mg FA/kg**•**diet and therefore added 0.5 and 1.0 mg extra FA/kg**•**diet, which is comparable in mice to consumption of one or two 400-μg FA tablets daily in people consuming a healthy diet.

Folate is essential for nucleotide synthesis, maintaining redox status, and supporting methylation reactions [10]. Because folate mediates Hcy re-methylation to methionine [29], folate deficiency raises Hcy concentrations [9]. Elevated Hcy levels increase oxidative stress [30] and amyloid-β-peptide accumulation, and they are associated with mild cognitive impairment [31]. FA may have antioxidant activity by functioning as a free radical scavenger [32] and low dose supplementation increases oxidative stress resistance and prolongs longevity in Caenorhabditis elegans [33]. There is some evidence that FA may decrease risk of neurodegenerative disease [34], stroke [35] and cardiovascular disease [36], all of which are related to oxidative stress.

Telomeric DNA damage may initiate genome instability and result in accelerated aging, cognitive decline, and neurodegenerative diseases [37]. Oxidative damage is a major type of DNA damage [38]. Owing to the high level of guanine in the 5'-TTAGGG-3' repeat sequence, telomeric DNA is particularly susceptible to oxidative damage. 8-OHdG is an oxidative product of deoxyguanosine and one of the most widely recognized biomarkers for detecting oxidative damage in DNA [39], and also represents the most frequent oxidative lesion in telomeric DNA [22]. Moreover, due to the telomeric heterochromatin state and its single stranded 3' overhang, oxidative damage in the telomere region is repaired with relatively low efficiently and may result in telomere shortening [40, 41], failing at protecting chromosomes from degradation, leading to cell senescence and death [25]. The present study found experimentally that FA supplementation that decreased ROS levels also alleviated telomeric DNA oxidation and telomere length shortening, indicating that alleviation of telomere attrition accompanied with decreased ROS levels might act as a potential mechanism in the process of delaying neurodegeneration and cognitive decline.

Additionally, we found the improved integrity of telomere was associated with improved mitochondrial biogenesis and function. Previously it had been shown that p53 activation induced by telomere dysfunction, in addition to classical p53-directed checkpoints of proliferative arrest and apoptosis, leads to deregulation of PGC expression [42]. Subsequently, through the PGC-1α–Nrf1–Tfam pathway, deregulated PGC-1α suppresses the transcription of Nrf1 and leads to decreased expression of Tfam, which is a potent stimulator of the duplication of mtDNA biogenesis [43]. Impaired mitochondrial biogenesis and function have deleterious effects on neuronal function, accelerate cellular senescence, and increase the risk of neurodegenerative disease [44, 45]. The present study demonstrated that a telomere-p53-mitochondria pathway may allow the improved telomere integrity to improve mitochondrial function and thereby prevent neuronal degeneration, which may further explain the protective effect of FA in delaying cognitive decline.

In conclusion, we demonstrate that FA supplementation delays age-related neurodegeneration and cognitive decline in SAMP8 mice, in which alleviated telomere attrition could serve as one influential factor in the process. These findings support the importance of sufficient folate consumption in older people for delaying cognitive decline.

## MATERIALS AND METHODS

### Animals and dietary treatment

All mice were provided by the Department of Laboratory Animal Science of Peking University Health Science Center. The Tianjin Medical University Animal Ethics Committee approved all the experimental procedures (TMUaMEC2019003).

Four-month-old male SAMP8 mice were randomly assigned to four treatment groups (15 mice/group): (1) FA-deficient diet (FA-D) group fed with FA-deficient diet and euthanized when 10 months old; (2) FA-normal diet (FA-N) group fed with FA-normal diet and euthanized when 10 months old; (3) low FA-supplemented diet (FA-L) group fed with low FA-supplemented diet and euthanized when 10 months old; (4) high FA-supplemented diet (FA-H) group fed with high FA-supplemented diet and euthanized when 10 months old. There was also an age-matched senescence-accelerated mouse resistant 1 (SAMR1) control group (Con-R), in which 4-month-old male SAMR1 mice were fed the FA-normal diet and euthanized when 10 months old. Finally, there was a young SAMP8 control group (Con-Y), in which 3-month-old male SAMP8 mice were fed the FA-normal diet and euthanized when 4 months old. All mice were housed in a specific pathogen-free facility under a controlled temperature (23±1)°C and humidity (50±5)% environment with a 12-h light/dark cycle, allowed ad libitum access to food and water.

The FA-deficient, FA-normal, low FA-supplemented and high FA-supplemented diets contained, respectively, <0.1, 2.0, 2.5 and 3.0 mg FA per kilogram of AIN-93M diet (Trophic Animal Feed High-Tech Co. Ltd, China). Compared with the FA-normal diet, the low and high FA-supplemented diet added 0.5 and 1.0 mg extra FA per kilogram of diet, which in mice is equivalent to people consuming a FA supplement of 400 or 800 μg daily that used for improving cognitive performance [13]. The FA-deficient diet was not fortified with FA, but it contained residual folate that was present inherently in its macro-ingredients and that could not be avoided completely. The concentration of this residual folate level in the FA-deficient diet was measured with the VitaFast FA kit (R-Biopharm AG, Germany) and found to be less than 0.1 mg per kilogram of diet.

Mice from the FA-D, FA-N, FA-L, FA-H and Con-R groups were euthanized when 10 months old, and mice from the Con-Y group were euthanized when 4 months old. After fasting overnight, all mice were euthanized with CO_2_. Blood was collected by cardiac puncture and then was centrifuged to obtain serum. Brain tissue, from which the cerebellum had been removed, was either fixed with 4% paraformaldehyde for pathological section staining and immunofluorescence staining to detect neurodegeneration and telomere length, or stored at −80°C after liquid nitrogen flash-freezing for biochemical, qPCR and western blot assays to determine ROS levels, telomeric attrition, PGC-1α–Nrf1–Tfam pathway expression and mitochondrial function.

### Folate assay

Folate levels in serum, RBC and brain were measured with an automated chemiluminescence system (Siemens Immulite 2000 Xpi, Germany) using a competitive protein binding assay, according to the manufacturer’s instructions. This system detected all types of folate with a detection sensitivity limit of 0.8 ng/mL. Serum samples were obtained as described in section *Animals and dietary treatment*. Whole blood samples were collected and hematocrit was determined by automated hematology analyzer (URIT-2900VetPLUS, China), then cells were lysed in freshly prepared 0.5% (w/v) ascorbic acid solution (1:26, v/v) for 3 h at RT (15-28 °C) in the dark, and centrifuged to obtain the supernatant that later was used for folate assay. The folate concentration in RBC was calculated approximately by multiplying the concentration in whole blood by 100/ hematocrit.

Brain tissue was isolated and crushed with liquid nitrogen, then the homogenate (1:10, w/v, diluted with ice-cold normal saline) was centrifuged to obtain the supernatant that later used for folate assay. The folate concentration in brain was normalized to the protein concentration that was determined by a bicinchoninic acid (BCA) protein quantitative kit (Boster Biological Technology, China).

### Measurement of Hcy and ROS levels

Serum samples were obtained as described above in section *Animals and dietary treatment*, and brain tissue was prepared as described above in section *Folate assay*. Hcy and ROS levels in brain tissue were normalized to the protein concentration that was measured using a BCA protein quantitative kit (Boster Biological Technology, China). ROS levels were indicated by H_2_O_2_ concentration, HO^**•**^ suppression ability and O_2_^**• –**^ suppression ability.

### Hcy assay

Serum Hcy concentration was quantified by an enzymatic cycling method. Serum samples were mixed with Hcy Reagent (Meikang Medical System, China) in a reaction cell, then the absorbance was measured at 340 nm by an Auto-Chemistry Analyzer (DIRUI Industrial Ltd, China) [46], with detection sensitivity limits of 0.33 μmol/L. For brain tissue, Hcy in the supernatant was measured by a homocysteine ELISA kit (Cell Biolabs, USA) according to the manufacturer’s instruction, with a detection sensitivity limit of 10 ng/mL.

### H_2_O_2_ assay

H_2_O_2_ in serum and brain tissue were analyzed with a H_2_O_2_ Assay Kit (Nanjing Jiancheng Bioengineering Institute, China) according to the manufacturer’s instructions. H_2_O_2_ reacts with ammonium molybdate to form a yellow complex that has an absorption peak at 405 nm. Therefore, H_2_O_2_ content was determined from the OD measured at 405 nm in a microplate reader (BioTek Instruments Inc, USA).

### HO^•^ suppression assay

The abilities of serum and brain tissue to suppress HO^**•**^ were measured with a Hydroxyl Free Radical Assay Kit (Nanjing Jiancheng Bioengineering Institute, China). The kit generates HO^**•**^ from 0.03% H_2_O_2_ reagent through the Fenton reaction and it develops red color with the electron transfer and color-developing reagents (Griess reagent). Absorbance at 550 nm is proportional to the amount of HO^**•**^ and is decreased by HO^•^ inhibitors. Therefore HO^•^ suppression was determined from the OD measured at 550 nm in a microplate reader (BioTek Instruments Inc, USA).

### O_2_^• –^ suppression assay

The abilities of serum and brain tissue to suppress O_2**• –**_ were measured with a Superoxide Anion Free Radical Assay Kit (Nanjing Jiancheng Bioengineering Institute, China). The kit generates O_2_^**• –**^ by the xanthine/xanthine oxidase reaction and it develops magenta color with the electron transfer and color-developing reagents (Griess reagent). Absorbance at 550 nm is proportional to the amount of O_2_^**• –**^ and is decreased by O_2_^**• –**^ inhibitors. Therefore O_2_^**• –**^suppression was determined from the OD measured at 550 nm in a microplate reader (BioTek Instruments Inc, USA).

### Behavioral tests

### MWM test

To evaluate spatial learning and memory abilities, MWM test was performed with the DMS-2 Morris water maze test system (Institute of Materia Medica at Chinese Academy of Medical Sciences, China). The MWM test has two phases, namely, an acquisition test and a spatial probe trial [47]. During the five consecutive days of the acquisition test, a mouse was released from an assigned starting point in each quadrant of the maze on each day’s test. If the mouse swam and climbed onto the platform within 60 s, its swimming time and distance were recorded as the latency of seeking escape. If the mouse failed to locate the platform within 60 s, it was guided to the platform, stayed there for 15 s, and latency was considered to be 60 s. The escape latency on each day was calculated as the average of 4 quadrants. After the final day of the acquisition test, the platform was removed and the spatial probe trial was carried out only once. Recorded during this trial were the time spent in the targeted quadrant and the number of times the mouse crossed the position where the platform previously had been located. Decreased value for escape latency indicates better spatial learning ability, and increased values for time spent in the targeted quadrant and number of crossings indicate better memory ability.

### OF test

To measure the locomotor activity and exploratory behavior in a novel environment [48], OF test was performed using the OFT-100 open field test system (Chengdu Taimeng Technology Co. Ltd, China). Before each test, the OF apparatus (50×50×50 cm^3^) was cleaned with a solution of 20% (v/v) ethanol. Then a mouse was placed in the center of the OF and allowed to explore spontaneously for 5 min. Performance was assessed under bright-lit conditions (~ 150 lx). Distance traveled and time spent in the center of the OF were measured.

### Histological assessment

A segment of brain was fixed with 4% (w/v) paraformaldehyde in 0.01 M PBS (pH 7.4) at 4 °C for 12 h, dehydrated and embedded in paraffin, and cut coronally into 5 μm slices by a rotary microtome (Leica, Germany). The slices were deparaffinized with xylene and rehydrated using a gradient of high-percentage ethanol to distilled water. Then HE staining, Nissl staining, and FJB staining were performed to assess neurodegeneration.

### HE staining

HE staining was performed with a HE Staining kit (Beyotime, China) according to the manufacturer’s instructions. Normal neurons have relatively big cell bodies with abundant cytoplasm and big round nuclei. In contrast, damaged neurons exhibited shrunken cell bodies with many empty vesicles and condensed nuclei. Images from cerebral cortex and hippocampal CA1 region (at ×200 magnification) were captured by an Olympus IX81 microscope (Olympus, Japan) in bright-field mode.

### Nissl staining

Nissl staining was performed to identify surviving neurons by visualizing the Nissl body in cytoplasm. Brain sections were stained with cresyl violet (Beyotime, China) according to the manufacturer’s instructions. Neurons with discernable and rich Nissl staining were counted as viable neurons, whereas neurons with lost Nissl bodies and condensed cytoplasm were considered as damaged neurons. Images from cerebral cortex and hippocampal CA1 region (at ×200 magnification) were captured by an Olympus IX81 microscope (Olympus, Japan) in bright-field mode, and the number of surviving neurons was analyzed with ImageJ software.

### FJB staining

To indicate the neurons that undergoing degeneration, FJB (Millipore, USA) staining was carried out according to a standard protocol [49]. In brief, after being deparaffinized and rehydrated, the slides were immersed in a 1% (w/v) sodium hydroxide contained 80% (v/v) ethanol solution for 5 min, transferred to 70% (v/v) ethanol, and distilled water. Next the slides were reacted with a solution of 0.06% (w/v) potassium permanganate for 10 min, rinsed with distilled water, and then immersed in a solution of 0.1% acetic acid (v/v) and 0.01% FJB (w/v, Millipore, USA) for 20 min. Finally, after rinsing with distilled water, the dried slides were mounted with 4',6-diamidino-2-phenylindole (DAPI, Vector Laboratories, USA). Images were captured using an FITC filter on an Olympus IX81 inverted fluorescence microscope system (Olympus, Japan). FJB-positive cells in cerebral cortex and hippocampal CA1 region (at ×200 magnification) were analyzed with ImageJ software.

### Measurement of 8-OHdG in genomic DNA and telomere attrition

### Measurement of 8-OHdG in genomic DNA

Total DNA in brain tissue was extracted using a DNA extraction kit (AidLab, China) and quantified with a NanoDrop 2000 instrument (Thermo Scientific, USA). Equivalent amounts of DNA were digested with a DNA digest mix that consisted of benzonase (Sigma-Aldrich, USA), phosphodiesterase I (Sigma-Aldrich, USA) and alkaline phosphatase (Sigma-Aldrich, USA) for 6 h at 37 °C [50]. Then the 8-hydroxy-2'-deoxyguanosine (8-OHdG) level in genomic DNA was determined using a DNA Damage ELISA Kit (StressMarq Biosciences, Canada) through a competitive enzyme immune assay, according to the manufacturer’s instructions. The absorbance at 450 nm was recorded using a microplate reader (BioTek Instruments Inc, USA).

### Measurement of oxidation level in telomeric DNA

Total DNA in brain tissue was extracted and quantified as described above in section *Measurement of 8-OHdG in genomic DNA*. Equivalent amounts of DNA were incubated with or without formamidopyrimidine DNA-glycosylase (FPG) (NEB, USA) overnight, followed by a telomere-specific PCR assay [51]. FPG cleaves oxidized base, hence incubation of oxidized guanine-containing DNA with FPG leads to a fragmentary template for the subsequent PCR and increases Ct values. Thus the difference between Ct values of FPG-treated versus untreated DNA represented the percentage of damaged DNA [52]. The telomere-specific PCR assay was performed with a thermal cycler condition of 95 °C for 2 min, followed by 40 amplification cycles (denaturation, 95 °C for 15 s; annealing and extension, 60 °C for 1min) and telomere primers (forward, 5'-cggtttgtttgggtttgggtttgggtttgggtttgg gtt-3'; reverse, 5'-ggcttgccttacccttacccttacccttacccttac cct-3'). All qPCR reactions were performed in a LightCycler 480 Ⅱ instrument (Roche Applied Science, Switzerland).

### Measurement of telomere length by qPCR

Total DNA in brain tissue was isolated and quantified as described above in section *Measurement of 8-OHdG in genomic DNA*. Telomere length was assessed by a qPCR method using an Absolute Mouse Telomere Length Quantification qPCR Assay Kit (ScienCell, USA) according to the manufacturer’s instructions. The telomere primer set recognized and amplified telomere sequences. A single copy reference primer set that recognizes and amplifies a 100 bp-long region on mouse chromosome 10 was used for data normalization. A reference genomic DNA sample with known telomere length served as a reference for calculating the telomere length of samples. Cycling conditions for both targets consisted of an initial 95 °C step for 10 min followed by 32 cycles (denaturation, 95 °C for 20 s; annealing, 52 °C for 20 s; and extension, 72 °C for 45 s). All qPCR reactions were performed in a LightCycler 480 II instrument (Roche Applied Science, Switzerland).

### Measurement of telomere length by telomere Q-FISH

Paraffin sections of brain were prepared as described above in section *Histological assessment*. After being deparaffinized with xylen and rehydrated using a gradient of high-percentage ethanol to distilled water, slides were immersed in citrate buffer (pH 6.0) in a steamer for 30 min. The cooled slides were immersed in 0.5% (v/v) Triton X-100 detergent for 20 min, transferred to PBS, and immersed in a gradient of 60% (v/v) ethanol to absolute ethanol. After drying at RT, 0.3 ng/μL Cy3-labeled telomere PNA probe (PNA Bio, Korea) hybridization solution was added to the slides. After denaturation (85°C, 5 min), the slides were left to hybridize overnight at RT in darkness. Next the slides were washed with Wash Solution Ⅰ(Formamide, PBS and blocking solution) and Wash Solution Ⅱ (1M Tris pH 7.4, 3M NaCl, Tween-20 and distilled H_2_O). Afterwards the slides were blocked with normal goat serum (Boster Biological Technology, China) for 1 h at RT, incubated with primary anti-NeuN antibody (1:200, Abcam, USA) overnight at 4 °C, washed with PBS, and incubated with secondary antibody (anti-mouse IgG fraction Alexa Fluor 488, 1:200, Proteintech, China) for 1 h at RT. Finally, the slides were counterstained with DAPI (Vector Laboratories, USA). DAPI staining was used to define nuclear area and quantify DNA content, and Cy3 staining was used to quantify telomere fluorescence. Data were analyzed using the Telometer (v2.1.4) to determine the intensity sums of all DAPI pixels (proportional to total nuclear DNA content) and Cy3 telomere pixels (proportional to telomere length) for each nucleus. Calculating the ratio of the telomere intensity sum to the corresponding DAPI intensity sum compensated for ploidy differences as well as for the variable fractions of nuclei present in the cutting plane of the tissue section [53].

### Regulation of telomere-p53-mitochondria pathway

To detect the regulation of telomere-p53-mitochondria pathway, mRNA and protein expressions of three mitochondrial biogenesis-related genes, including PGC-1α, Nrf1 and Tfam were quantified by qPCR and western blot assays. Additionally, phosphorylation level at Ser15 of p53 protein was also quantified by western blot.

Total RNA in brain tissue was extracted using an Eastep Super Total RNA Extraction Kit (Promega, USA) and quantified by NanoDrop 2000 instrument (Thermo Scientific, USA). 1 μg of total RNA was used to synthesize the first-strand cDNA with an All-in-One First-Strand cDNA Synthesis Kit (GeneCopoeia, USA) according to the manufacturer’s instructions. Primers (GenScript, China) were specific for PGC-1α (forward, 5′- ccctgccattgttaagacc -3′; reverse, 5′- tgctgctgttcctgttttc -3′), Nrf1 (forward, 5′- agcacggagtgacccaaac -3′; reverse, 5′- tgtacgtggctacatggacct -3′), Tfam (forward, 5′- attccgaagtgt ttttccagca -3′; reverse, 5′- tctgaaagttttgcatctgggt -3′) and β-actin (forward, 5′- gacatggagaagatctggca -3′; reverse, 5′- ggtctcaaacatgatctgggt -3′). The GoTaq qPCR Master Mix (Promega, USA) was added into a 20-μL PCR reaction mix that containing cDNA and primers. The qPCR assay was performed with a thermal cycler condition of 95 °C for 2 min, followed by 40 amplification cycles (denaturation, 95 °C for 15 s; annealing and extension, 60 °C for 1min). The housekeeping gene β-actin was used for data internal normalization. All qPCR reactions were performed in a LightCycler 480 II instrument (Roche Applied Science, Switzerland).

Brain tissue extracts were prepared in cell lysis buffer (Beyotime, China) with a motor-driven tissue homogenizer (PT1200E, Switzerland), and the protein concentration was determined with a BCA protein assay kit (Boster Biological Technology, China). Equivalent amounts of extracted protein were separated on 12% sodium dodecyl sulfate-polyacrylamide gel by electrophoresis, and then were transferred to PVDF membranes (Merck Millipore, USA). After blocking with 5% (w/v) bovine serum albumin for 1 h at RT, the membranes were incubated with rabbit anti-p53 antibody (1:1000, Proteintech, China), rabbit anti-phosphorylated p53 at Ser15 (1:1000, Bioworld Technology, USA), rabbit anti-PGC-1α (1:1000, Proteintech, China), rabbit anti-Nrf1 (1:1000, Proteintech, China), rabbit anti-Tfam (1:1000, Proteintech, China), and rabbit anti-β-actin (1:2000, Proteintech, China) overnight at 4 °C. After washing with TBST, immunoblots were incubated with Horseradish Peroxidase-conjugated goat anti-rabbit IgG antibody (1:2000, Proteintech, China) for 1 h at RT. Finally, the immunoblots were visualized using chemiluminescence reagent (Merck Millipore, USA) and a ChemiDoc XRS+ Imaging System (Bio-Rad, USA). The intensity of each protein band was quantified by ImageJ software and normalized to the respective β-actin band.

### Measurement of mitochondrial biogenesis and function

### Measurement of relative mtDNA copy number

Total DNA was extracted from brain tissue using a DNA extraction kit (AidLab, China). Relative mtDNA copy number was determined by a qPCR method with the GoTaq qPCR Master Mix (Promega, USA) [54]. Mitochondrial gene ND1 and nuclear gene hexokinase 2 (HK2) were measured to represent mtDNA and nDNA, respectively, and their ratio (mtDNA/nDNA) was calculated to quantify mtDNA copy number. The thermal cycler condition was 95 °C for 5 min, followed by 40 amplification cycles (denaturation, 95 °C for 10 s; annealing 60 °C for 10 s; and extension, 72 °C for 20 s). Primers were specific for ND1 (forward, 5′-ctagcagaaa caaaccgggc-3′; reverse, 5′-ccggctgcgtattctacgtt-3′) and HK2 (forward, 5′-gccagcctctcctgattttagtgt-3′; reverse, 5′-gggaacacaaaagacctcttctgg-3′). All qPCR reactions were performed with a LightCycler 480 II instrument (Roche Applied Science, Switzerland).

### Measurement of mtDNA deletions

Total DNA was extracted from brain tissue using a DNA extraction kit (AidLab, China). Multiplex qPCR assay was used to quantify mtDNA deletions. Since mitochondrial gene ND4 is located in the major arch of the mtDNA where deletion frequently occurs, and mitochondrial gene ND1 is rarely deleted, the ratio between non-deleted fraction of mtDNA (ND4) and the total amount of mtDNA (ND1) was calculated to quantify mtDNA deletion level [55]. The TaqMan probe method was carried out using the following primers and probes (GenScript, China): ND1(forward, 5′-atatcctaacactcctcgtcc-3′; reverse, 5′-agggccttttcgtagttg-3′; probe, 5′-6FAM-ttctaatcgccatagccttcctaac-BHQ1-3′); ND4 (forward, 5′-aatatattctcctcagacccc-3′; reverse, 5′-aggtggttttggctagct-3′; probe, 5′-VIC-ccacaccattaattattt taagagcctg-BHQ1-3′). The GoldStar TaqMan Mixture (CoWin Biosciences, China) was added into a 20 μL reaction mix with probe and primer concentration at 250 and 300 nM, respectively. The thermal cycler condition was 2 min at 50 °C, 10 min at 95 °C, and 40 amplification cycles (denaturation: 95 °C for 15 s; annealing and extension: 60 °C for 1 min). qPCR was performed with a LightCycler 480 II instrument (Roche Applied Science, Switzerland).

### Measurement of ATP content

Mitochondrial function in brain tissue was assessed by ATP content using an Enhanced ATP Assay Kit (Beyotime, China) according to the manufacturer’s instructions. ATP-derived luminescence was measured with a luminometer microplate reader (BioTek Instruments Inc, USA). The tissue ATP level was normalized to the protein concentration that was determined by a BCA protein assay kit (Boster Biological Technology, China).

### Statistical analysis

Results were expressed as mean ± standard deviation (SD). At the end of the study, n=10 mice/group was selected based on simple random sampling method for subsequent assays. Comparisons among different groups were performed by one-way ANOVA and followed by Student–Newman–Keuls test for multiple comparisons. Repeated measures data of escape latency in MWM were compared using the repeated-measures ANOVA. The statistical software package SPSS 24.0 (IBM Corp., USA) was used to evaluate differences among groups, which were considered statistically significant at *P*-value <0.05.
